# The extension of total gain (TG) statistic in survival models: properties and applications

**DOI:** 10.1186/s12874-015-0042-x

**Published:** 2015-07-01

**Authors:** Babak Choodari-Oskooei, Patrick Royston, Mahesh K.B. Parmar

**Affiliations:** MRC Clinical Trials Unit at UCL, Aviation House, 125 Kingsway, London, WC2B 6NH UK

**Keywords:** Total gain, Predictive ability, Cox proportional hazards model, Non-proportional hazards, Time-dependent covariate

## Abstract

**Background:**

The results of multivariable regression models are usually summarized in the form of parameter estimates for the covariates, goodness-of-fit statistics, and the relevant p-values. These statistics do not inform us about whether covariate information will lead to any substantial improvement in prediction. Predictive ability measures can be used for this purpose since they provide important information about the practical significance of prognostic factors. *R*^2^-type indices are the most familiar forms of such measures in survival models, but they all have limitations and none is widely used.

**Methods:**

In this paper, we extend the total gain (*TG*) measure, proposed for a logistic regression model, to survival models and explore its properties using simulations and real data. *TG* is based on the binary regression quantile plot, otherwise known as the predictiveness curve. Standardised *TG* ranges from 0 (no explanatory power) to 1 (‘perfect’ explanatory power).

**Results:**

The results of our simulations show that unlike many of the other *R*^2^-type predictive ability measures, *TG* is independent of random censoring. It increases as the effect of a covariate increases and can be applied to different types of survival models, including models with time-dependent covariate effects. We also apply *TG* to quantify the predictive ability of multivariable prognostic models developed in several disease areas.

**Conclusions:**

Overall, *TG* performs well in our simulation studies and can be recommended as a measure to quantify the predictive ability in survival models.

**Electronic supplementary material:**

The online version of this article (doi:10.1186/s12874-015-0042-x) contains supplementary material, which is available to authorized users.

## Background

Predictive ability measures are useful in medical practice as well as biomedical research. Measures that quantify the degree of association between the dependent and explanatory variables in a model are one class of such measures. In this class, *R*^2^ is the most familiar predictive ability measure for the linear regression model *E*(*Y*|*Z*)=*β*^′^*Z* where *Z* and *β* are a set of covariate and parameter vectors, respectively. *R*^2^ is the proportion of variability in the outcome that is explained through the covariates in the model, where variability is measured by the variance of the outcome variable

(1) $$ R^{2}=\frac{Var(Y)-E(Var(Y|Z))}{Var(Y)}.   $$

To estimate *R*^2^ in simple linear regression, *Var*(*Y*) and *E*(*Var*(*Y*|*Z*)) can be replaced with the (scaled) estimates of *SST* (total sum of squares) and *SSE* (residual sum of squares), respectively. *R*^2^ has several appealing properties, of which the most important are: *i*) *R*^2^∈ [0,1]: it lies between 0 (representing no predictive ability) and 1 (perfect predictive ability); *ii*) monotonicity: it increases with the size of the covariate effect, ∥*β*∥, in the model; and *iii*) interpretability as the percentage of variability in the outcome that is explained by the covariates [1].

Due to its popularity, analogous *R*^2^-type statistic have been developed for other regression models [2], including logistic and survival models [1, 3]. Logistic regression has wide applications in medical research. The response variable in this model is a binary variable *Y*, which takes the value 1 for those experiencing the event of interest, e.g. cases, and 0 for others, e.g. controls. In this model, the mean of *Y*, the probability of experiencing the event, is *π*. The model is represented by *logit*(*π*|*Z*)=*β*^′^*Z*. Many *R*^2^ counterparts have been proposed for use in logistic regression [2]. Since the predictions for the outcome variable are expressed as event probabilities in this model, different functions have been proposed to replace *Var*(*Y*) and *E*(*Var*(*Y*|*Z*)) in Equation 1. One such example is the (expected) Brier scores [4] under the null and the model with covariate *Z*.

For a logistic model, discrimination measures [5, 6] can be regarded as an alternative class of predictive ability measures (see Table one in [6]). The *c*-statistic [7] belongs to this class which has been extended to survival models. The *c*-index is identical to the area under the receiver operating characteristic (*ROC*) curve [6]. It can be interpreted as the chance that a case will have a higher predicted probability of event occurrence than a control. The *c*-statistic is a rank-order statistic for predictions against true outcomes, and ranges between 0.5 (no discrimination) to 1 (perfect discrimination).

In 1999, Copas [8] proposed a new approach to summarise the predictive ability of a logistic regression model. The logit rank plot is based on the cumulative distribution function of the prognostic index (PI) *β*^′^*Z*. Later, Bura and Gastwirth [9] proposed the binary regression quantile plot, also known as predictiveness curves [10]. A predictiveness curve displays the distribution of estimated (or predicted) event probabilities versus their quantiles. Bura and Gastwirth [9]’s approach differs from the receiver-operating characteristic (*ROC*) curve and the logit rank plot of Copas [8] as it does not classify subjects into high risk or low risk classes. Bura and Gastwirth [9] extended the plot and proposed a new measure of predictive ability, named total gain (*TG*), for a logistic regression model. *TG* is defined as the integrated absolute difference between the predicted event probabilities and the ‘average’ event probability over the cumulative distribution function of the PI. Bura and Gastwirth [9] also proposed a standardised counterpart *TG*_*STD*_ which, similar to *R*^2^ in linear regression, lies between 0 and 1. Although, in principle, Bura and Gastwirth’s measures can be immediately applied to survival data, their properties have not been investigated in survival data where censoring is present.

Many analogous *R*^2^-type statistics have been proposed for the survival models [11]. Some of the measures are only defined for the Cox proportional hazards (PH) model [12, 13], and some have been generalised for use with more general types of survival models [14, 15]. However, as has been shown by Choodari-Oskooei *et al.* [1, 3] and others [16], they all have shortcomings. The adverse effect of censoring on most of the measures is one of the main reasons for this. Nonetheless, based on their comprehensive empirical investigations, Choodari-Oskooei *et al.* [1, 3] recommended a set of measures for practical application. They are $R_{\textit {PM}}^{2}$, ${R_{D}^{2}}$, and ${\rho _{W}^{2}}$ - see Additional file 1 for their definition. These statistics quantify the amount of prognostic information resulting from the model and provide an overall measure of predictive ability for the whole follow-up period. Also, Graf *et al.* [14] proposed $ R_{\textit {BS}}^{2}(t)$ which uses the (time-dependent) marginal and conditional Brier scores to replace *Var*(*Y*) and *E*(*Var*(*Y*|*Z*)) in Equation 1 - see Additional file 1. $R_{\textit {BS}}^{2}(t)$ quantifies the accuracy of (survival) probability predictions at the individual level at a particular time-point. Among the above four measures, $R_{\textit {BS}}^{2}(t)$ is the only statistic that can explicitly assesses the model’s (predictive) performance at any time point over the follow-up period. In their current form, $ R_{\textit {PM}}^{2}$, ${R_{D}^{2}}$, and ${\rho _{W}^{2}}$ are unsuitable for this purpose, hence their application is limited. For example, they can not be applied to models with time-dependent covariate effects included.

The purpose of the present article is fourfold. First, we extend the predictiveness curve, the *TG* statistic, and its counterpart *TG*_*STD*_ to survival models. Second, we explore their properties in survival models using extensive simulation studies. Third, we show the relationship between a (version of) total gain measure which is based on the squared error loss function with the Schemper’s *V*-measure [17] for binary outcomes and $R_{\textit {BS}}^{2}$ for survival models. Fourth, we discuss the application of *TG* in prognostic modelling and compare its estimates to the those of other recommended measures using real data. We also show that both *TG* and *TG*_*STD*_ explicitly assess the performance of the model at a specific time point over the follow-up period.

The structure of the paper is as follows. In “Methods”, we describe the predictiveness curve and the *TG* statistic for a logistic regression model. In “Extension to survival models”, we propose our extension to survival models. We use a real data set from breast cancer to illustrate the steps that should be taken to draw the predictiveness curve, and also to estimate both *TG*(*t*) and *TG*_*STD*_(*t*) for a survival model. In “Results”, we present the results of our simulation studies to explore the performance of the proposed measure(s) for survival models under numerous scenarios. We study the impact of censoring, covariate distribution, influential (extreme and outlier) observations, and non-proportional hazards (non-PH) on the measure. We also investigate the monotonicity property of the measure as well as the effect of categorising continuous prognostic factors. In “Applications”, we apply our proposed measures to real data from several studies, and compare the results to those from other recommended *R*^2^-type measures. Finally, we discuss the findings and make recommendations in “Discussion”.

## Methods

### Total gain (TG) measure

The total gain (*TG*) measure [9] is based on the predictiveness curve [10]. We first describe this curve in a logistic regression model. We then extend the plot and present an analogous *TG* measure for survival models.

#### Predictiveness curve in logistic regression

Let *Y* denote a binary outcome variable *Y*∈{0,1}, such as incidence of disease or occurrence of an event within a specified time period and let *Z* denote a set of prognostic factors (or covariates) used to predict the outcome. For example, elements of the Framingham risk score (age, gender, total and high-density lipoprotein cholesterol, systolic blood pressure, treatment for hypertension and smoking) have been used in logistic regression to predict occurrence of a cardiovascular event (http://hp2010.nhlbihin.net/atpiii/calculator.asp). In the multivariable logistic regression model, we can estimate the predicted risk associated with the value (of prognostic factors) *Z*=*z* as *π*|*Z*=Pr [*Y*=1|*Z*=*z*]. Huang *et al.* [10] defined the predictiveness curve as the distribution of estimated risk over the cumulative distribution of the PI of the model. Let *υ*=*F*(*β*^′^*Z*) where *F*(.) is the cumulative distribution function. In other words, *υ* represents the proportional rank of PI across its values from the smallest to the largest. The predicted risk associated with *υ*∈ [0,1] is defined as

$$R(\upsilon)=\text{Pr}\, [Y=1|\upsilon ]. $$

In practice, *β* is estimated with $\widehat {\beta }$ in which case $ \widehat {\pi }|Z=\text {Pr}\,[Y=1|\widehat {\beta }]$, $\widehat {\upsilon }=F(\widehat {\beta }z)$, and $\widehat {R}(\upsilon)=\text {Pr}\, [Y=1|\widehat {\upsilon }]$. In effect, the predictiveness curve is a plot of the risk *R*(*υ*) versus the (scaled) ranks of PI. Plotting the risks against the ranks of PI enables us to compare different risk scores from different models as all score values are being transformed to a common scale, i.e. between 0 and 1. Another property of the plot is that it remains invariant to monotonic transformation of the PI - all that matters is that Pr [*Y*=1|*Z*=*z*] is an increasing function of the PI.

In logistic regression, the estimated risks are a monotonic function of the PI. Therefore, the curve is in effect a P-P plot of the cumulative distribution function of the estimated risks themselves. This gives the curve a useful interpretation as it shows the proportion *υ* of individuals in the study with estimated risks less than *R*(*υ*).

**Fig. 1:**
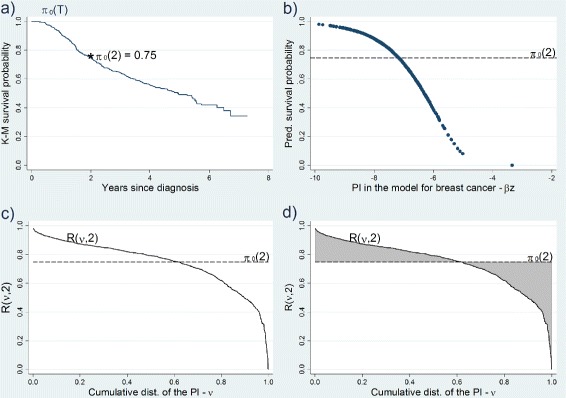
Predictiveness curve, *T* *G*(*t*) and *T* *G*_*STD*_(*t*) at 2 years in the breast cancer study. **a** the Kaplan-Meier survival estimates over time,$\widehat {\pi }_{0}(t);$ **b** plot of predicted survival probabilities $ S(t^{\ast }|z;\widehat {\beta })$ vs the PI of the model; **c** the predictiveness curve *R*(*υ*;*t* ^∗^) at *t* ^∗^=2; **d** *T* *G*(*t*) is the shaded area - see text and Additional file 2 for the prognostic factors included in the model

Let *π*_0_=Pr [*Y*=1] denote the overall prevalence of disease or occurrence of an event. Its estimate $\widehat {\pi }_{0}$, which can be regarded as the estimate of the ‘average’ event probability for all individuals, can obtained from the null model. For a completely ‘uninformative’ prognostic factor, the predictiveness curve is a horizontal line *R*(*υ*)=*π*_0_ because in this scenario *π*|*Z*=Pr [*Y*=1|*Z*=*z*]=Pr [*Y*=1]=*π*_0_ for all individuals. On the other hand, a perfect prognostic factor assigns *π*|*Z*=1 for the proportion *π*_0_ of subjects with *Y*=1 and *π*|*Z*=0 for the proportion 1−*π*_0_ with *Y*=0. The predictiveness curve in this scenario is the step function *R*(*υ*)=*I*[(1−*π*_0_)<*υ*], where *I*[ ·] is the indicator function. For essentially all recognised prognostic factors, the plot lies between these two extremes. We will illustrate this in Fig. 1 of Section “Extension to survival models” when we present our extension to survival models.

#### TG in logistic regression

The *TG* statistic can be directly visualised from the predictiveness curve. It is a non-negative, unitless measure of the total cumulative distance between the average risk probability, *π*_0_, and the estimate of risk over the cumulative distribution function of the PI

(2) $$ TG={\int_{0}^{1}}\left\vert R(\upsilon)-\pi_{0}\right\vert d\upsilon.   $$

Bura and Gastwirth [9] showed that *TG* has an upper bound of 2*π*_0_(1−*π*_0_). This can be used as a ‘scaling’ factor to standardise the measure

(3) $$ TG_{STD}=\frac{TG}{2\pi_{0}(1-\pi_{0})}   $$

so that, similar to the other analogous *R*^2^-type measures, *TG*_*STD*_∈ [0,1].

Based on the normal approximation, Bura and Gastwirth [9] developed a (complex) asymptotic formula for the variance of *TG* in logistic regression - see Additional file 1. The formula is based on the normal approximation to $\widehat {\pi }$. For this reason, the proposed (asymptotic) variance formula might not provide a good approximation if the (effective) sample size is small and $\widehat {\pi }$ is near 0 or 1. However, bootstrap resampling can be used for this purpose in both small and large sample sizes.

#### Relationship to Brier score and Schemper’s *V*

For a binary outcome, the standardised total gain statistic in Equation 3 uses the mean absolute deviation (i.e. an average *L*^1^-norm function) between the model-based predicted risk probabilities (*π*|*Z*) and the average risk *π*_0_ to provide a measure of predictive ability. Mean squared deviation (i.e. an average *L*^2^-norm function) is an alternative loss function that can be used to define *TG*_*STD*_. In fact, Pepe *et al.* [18] proposed the following *R*^2^-type measure using the squared error loss function

(4) $$ R_{Pepe}^{2}=\left[\pi_{0}(1-\pi_{0})\right]^{-1}{\int_{0}^{1}}(R(\upsilon)-\pi_{0})^{2}d\upsilon.   $$

It can be shown that (see Additional file 1) in a correctly-specified model, $R_{\textit {Pepe}}^{2}$ becomes identical to the Schemper *V*-measure [17] based on the Brier score for a binary outcome

(5) $$ V_{B}=\frac{{\sum_{i}^{n}}(Y_{i}-\pi_{0})^{2}-{\sum_{i}^{n}}(Y_{i}-\pi_{i}|Z)^{2}}{\sum_{i}^{n}(Y_{i}-\pi_{0})^{2}}   $$

where *n* is the sample size. Mittlbock and Schemper [19] studied *V*_*B*_ for a logistic model and Graf *et al.* [14] proposed a modified version of it for survival models, i.e. $R_{\textit {BS}}^{2}$ - see Additional file 1.

### Extension to survival models

In this section, we extend the predictiveness curve and the *TG* statistic to a survival model with a focus on the Cox PH regression model.

#### Model and notation

In a survival study with *n* subjects, denote by *T*_*i*_ and *C*_*i*_ (*i*=1,2,⋯,*n*) the failure and censoring times. For individual *i* we observe *X*_*i*_= min(*T*_*i*_,*C*_*i*_), and *δ*_*i*_=*I*(*T*_*i*_≤*C*_*i*_). The Cox PH model assumes that the conditional hazard function

$$h(t|Z;\beta)=h_{0}(t)\mathit{\exp}(\beta^{\prime}Z) $$

where *t* is a non-negative random variable denoting the time to a failure event, and *h*_0_(*t*) is the baseline hazard. For this model, $S_{0}(t)=\exp \left \{ -{\int _{0}^{t}}h_{0}(u)du\right \} $ represents the baseline survival function, and $S(t|Z;\beta)=S_{0}(t)^{\exp (\beta ^{\prime }Z)}$ is the survival function for the model with covariate vector *Z*.

#### Predictiveness curve and TG for a survival model

In prognostic studies of survival data, we are generally interested in the accuracy of the predictions in terms of survival probabilities. For this reason, we define the predictiveness curve and *TG* and *TG*_*STD*_ statistics based on the (predicted) survival probabilities from a fitted model. This means *R*(*υ*) is constructed from (the estimates of) *S*(*t*|*Z*;*β*) in which case *π*_0_ is replaced with the Kaplan-Meier survival estimate at time *t*, $\widehat {\pi }_{0}(t)$. The corresponding predictiveness curve is a function of time. Therefore, a time-dependent predictiveness curve at time *t* is defined as

(6) $$ R(\upsilon ;t)=\text{Pr}[T>t|\upsilon ]   $$

where *υ*=*F*(*β*^′^*Z*) is the cumulative distribution function - i.e. *υ*∈[0,1] is the proportional rank of PI across its values from the smallest to the largest. The corresponding time-dependent *TG*(*t*) measure at time *t* is

(7) $$ TG(t)={\int_{0}^{1}}\left\vert R(\upsilon ;t)-\pi_{0}(t)\right\vert d\upsilon.   $$

Similar to the logistic regression case, it can be shown that *TG*(*t*) has an upper bound of 2*π*_0_(*t*)(1−*π*_0_(*t*)) [9]. Therefore,

(8) $$ TG_{STD}(t)=\frac{TG(t)}{2\pi_{0}(t)(1-\pi_{0}(t))}   $$

In practice, *β* is estimated with $\widehat {\beta }$ in which case $ \widehat {\pi }(t)|Z=\Pr [T>t|\widehat {\beta }]$, $\widehat {\upsilon }=F(\widehat {\beta }z)$, and $\widehat {R}(\upsilon ;t)=\text {Pr}\,[T>t|\widehat {\upsilon }]$.

The Cox PH regression model has been used to develop a multivariable prognostic model for breast cancer [20]. The proposed model, which includes several factors, is based on a cohort study by the German Breast Cancer Study Group in primary node positive breast cancer [21] - see “Applications” and Additional file 2 for further details. Figure 1(a) shows the Kaplan-Meier plot of recurrence-free survival probabilities *π*_0_(*t*) for this study. We use this data set to illustrate the steps that should be taken to estimate both *TG*(*t*) and *TG*_*STD*_(*t*):

1. Choose a clinically relevant time point *t*^∗^, e.g. 2 years in the breast cancer study [20].
2. Fit the model with covariate vector *Z* and obtain the predicted survival probabilities given covariate vector *Z* at time *t*^∗^, i.e. $ S(t^{\ast }|z;\widehat {\beta })$. The Kaplan-Meier estimate of survival for all individuals at time *t*^∗^ should also be obtained, i.e. $\widehat { \pi }_{0}(t^{\ast })$ - Fig. 1(a).
3. Plot the estimates of survival probabilities from the model $S(t^{\ast }|z;\widehat {\beta })$ against the PI, i.e. Fig. 1(b).
4. Replace the actual values of the PI with its proportional ranks *υ* - see Fig. 1(c). This is the predictiveness curve for the survival probability predictions at *t*^∗^=2 years. In this graph the dashed line represents $\widehat {\pi }_{0}(t^{\ast })$, i.e. the Kaplan-Meier survival estimate at *t*^∗^=2 years, and the solid curve is the predictiveness curve for the model with covariate vector *Z*.
5. The shaded area between the solid curve and the dashed line in Fig. 1(d) is *TG*(*t*^∗^) and can be considered as the gain in terms of predictive ability when using prognostic factors *Z* compared with not using them.
6. $\widehat {TG}_{\textit {STD}}(t^{\ast })$ is the ratio of the area between the solid curve and the dashed line, i.e. $\widehat {TG}(t^{\ast })$, to $2 \widehat {\pi }_{0}(t)(1-\widehat {\pi }_{0}(t))$.

In this example, $\widehat {TG}(2)$ and $\widehat {TG}_{\textit {STD}}(2)$ are 0.13 (95 % bootstrap CI: 0.11-0.15) and 0.33 (95 % bootstrap CI: 0.29-0.38), respectively.

## Results

### Simulation study

We conducted extensive simulation studies to explore the properties of *TG*(*t*) and *TG*_*STD*_(*t*). Choodari-Oskooei *et al.* [1] described the properties that a ‘good’ measure of predictive ability for a survival model should possess. They are: *i*) independence from censoring; *ii*) monotonicity; *iii*) robustness against influential (extreme and outlier) observations; and *iv*) interpretability. Our simulations, therefore, were carried out to explore the performance of the measures with respect to these criteria.

In this section, we first describe the simulation model. Then, we present the results of simulations and assess the performance of the measures with respect to the above-mentioned criteria. We investigate the upper bound of both *TG*(*t*) and *TG*_*STD*_(*t*), as well as the impact of non-proportional hazards on *TG*_*STD*_(*t*). The simulation model (exponential), censoring mechanisms (random censoring), censoring proportions, covariate distributions (normal, positively skewed, and negatively skewed), and covariate effects assumed in our studies are explained below.

#### Simulation of censored time-to-event data

We simulated time-to-event data from the exponential distribution with baseline hazard rate *λ*. The survival time in a proportional hazards model with a covariate, *Z*, was simulated as

(9) $$ T=\frac{-\ln (U)}{\lambda}\exp (-\beta Z)   $$

where *U* is sampled from the standard uniform distribution, *U*(0,1). To generate randomly censored survival times, we followed guidelines provided by Burton *et al.* [22].

#### Design parameters

*Covariate distribution and effects*: We study the measures in the context of multiple regression where the PI, i.e. the linear predictor, in the model is generally a function of several variables. As a result of the central limit theorem [1], the prognostic index should tend to Normality as the dimension of the parameter vector *β* increases. However, skewed prognostic factors are not uncommon in medical research - for example see the distribution of the number of positive lymph nodes (skewness: 2.8 and Kurtosis: 16.2) and progesterone receptor (skewness: 4.8 and Kurtosis: 37.8) in the breast cancer data set studied in [1]. Thus, we conducted our simulation study for three covariate distributions: normal *N*(0,1); negatively skewed with skewness of −2.8; and positively skewed with skewness of 2.8. We applied the method proposed by Fleishman [23] to transform the standard normal distribution to skewed distributions with mean 0 and variance 1. For all covariate distributions, we carried out our simulations under four covariate effects of exp(*β*)={1.25,1.5,2,4}.

*Censoring mechanisms*: we carried out our simulations under both random and type I (or administrative) censoring with 20 *%*, 50 *%*, and 80 *%* censoring proportions. Since the results were very similar, we only present the results under the random censoring condition.

*Sample size and the number of replicates*: sample size was set at 500 individuals, and the number of replicates was 5,000 is all experimental conditions.

### Uncensored data

In this section, we present the results for *TG*_*STD*_(*t*) and discuss those for *TG*(*t*) in uncensored data. The results of simulation studies to evaluate the means of *TG*_*STD*_(*t*) under 3 covariate distributions and 4 covariate effects are presented in Table 1. In the simulations, we considered 6 time points to show the behaviour of the measure over time. The time points are the 5 th, 10th, 15th, 20th, 25th, and 50th centile of the exponential distribution used to generate the survival times. They correspond to 6 time points as *T*_1_=2.57, *T*_2_=5.28, *T*_3_=8.17, *T*_4_=11.20, *T*_5_=14.43, and *T*_6_=34.66.

**Table 1:** Mean and standard deviation (in brackets) of *T* *G*_*STD*_(*t*) at 6 different time points by the covariate distribution (Cov.) and covariate effect (*e* *x* *p*(*β*)) - sample size is 500, and 0 % censoring

Cov.	exp(*β*)	TG _*STD*_(*T*_1_)	TG _*STD*_(*T*_2_)	TG _*STD*_(*T*_3_)	TG _*STD*_(*T*_4_)	TG _*STD*_(*T*_5_)	TG _*STD*_(*T*_6_)
	1.25	0.090 (0.019)	0.093 (0.020)	0.095 (0.020)	0.098 (0.021)	0.101 (0.021)	0.121 (0.025)
Normal	1.5	0.163 (0.020)	0.168 (0.021)	0.172 (0.021)	0.177 (0.022)	0.182 (0.022)	0.216 (0.025)
	2	0.274 (0.022)	0.281 (0.022)	0.288 (0.023)	0.295 (0.023)	0.303 (0.023)	0.346 (0.024)
	4	0.499 (0.025)	0.505 (0.023)	0.511 (0.022)	0.517 (0.021)	0.523 (0.021)	0.558 (0.020)
	1.25	0.093 (0.023)	0.094 (0.23)	0.095 (0.023)	0.096 (0.023)	0.097 (0.022)	0.107 (0.023)
Pos.	1.5	0.187 (0.032)	0.184 (0.029)	0.182 (0.027)	0.181 (0.026)	0.181 (0.025)	0.185 (0.022)
skewed	2	0.347 (0.041)	0.325 (0.034)	0.313 (0.031)	0.304 (0.028)	0.298 (0.026)	0.284 (0.022)
	4	0.603 (0.035)	0.557 (0.031)	0.529 (0.029)	0.509 (0.027)	0.494 (0.026)	0.449 (0.023)
	1.25	0.070 (0.014)	0.072 (0.015)	0.075 (0.016)	0.078 (0.016)	0.081 (0.017)	0.101 (0.022)
Neg.	1.5	0.118 (0.014)	0.122 (0.015)	0.127 (0.016)	0.132 (0.016)	0.138 (0.017)	0.176 (0.022)
skewed	2	0.180 (0.015)	0.188 (0.015)	0.196 (0.016)	0.205 (0.017)	0.215 (0.018)	0.280 (0.024)
	4	0.289 (0.016)	0.307 (0.017)	0.326 (0.018)	0.346 (0.019)	0.367 (0.020)	0.491 (0.025)

Generally, the estimates of the measure are higher in positively skewed covariates and lower in negatively skewed covariates. In most scenarios, there is a mild increase with increasing time. We also conducted further simulations beyond the time point *T*_6_ and to the maximal time points (data not shown). The results showed that *TG*(*t*) is 0 at time 0. It increases until a certain point (i.e. median of the underlying time-to-event distribution), and then decreases again towards 0 at the maximal time points. However, *TG*_*STD*_(*t*) does not follow this pattern and its trend over time depends on the size of the effect (and distribution) of the covariate. In all scenarios, the estimates of *TG*(*t*) and *TG*_*STD*_(*t*) increase with increasing covariate effects. We also carried out similar simulations with sample sizes of 200 and 1000 which resulted in similar conclusions. The results showed that the dispersion of the measures decreases as sample size increases, as expected.

Furthermore, we carried out similar simulation studies on the time-dependent version of $R_{\textit {Pepe}}^{2}$ where *R*(*υ*), and *π*_0_ in Equation 4 are replaced with the corresponding *R*(*υ*;*t*), and *π*_0_(*t*) - data not shown. Our results confirmed the underlying theory that $ R_{\textit {Pepe}}^{2}(t)$ and $R_{\textit {BS}}^{2}(t)$ are asymptotically the same. However, since $R_{\textit {BS}}^{2}(t)$ is a non-parametric measure, its sampling distribution has larger variance. For example, for *HR*= 4 with one normally distributed covariate the means (standard deviation) of $R_{\textit {Pepe}}^{2}(T_{6})$ and $R_{\textit {BS}}^{2}(T_{6})$ are 0.40 (SD: 0.02) and 0.40 (SD: 0.04), respectively.

### Censoring effect

In this section, we present the results of simulations to study the impact of random censoring on *TG*_*STD*_(*t*). The results are demonstrated in Table 2. The simulations were carried out for three covariate distributions, three censoring proportions (20 *%*, 50 *%*, and 80 *%*), and one covariate effect of 0.693 (exp(*β*)=2). We report the average percentage difference between the means of the measures in the censored data and the corresponding means of the measures in the uncensored condition, i.e. the percentage bias.

**Table 2:** The percentage difference in the means of *T* *G*_*STD*_(*t*) in censored data from those of *T* *G*_*STD*_(*t*) in the corresponding uncensored data by covariate distribution (Cov.), and censoring proportion

Cov.	*%*cen.	*T* *G*_*STD*_(*T*_1_)	*T* *G*_*STD*_(*T*_2_)	*T* *G*_*STD*_(*T*_3_)	*T* *G*_*STD*_(*T*_4_)	*T* *G*_*STD*_(*T*_5_)	*T* *G*_*STD*_(*T*_6_)
	20	0.1	0.1	0.1	0.1	0.1	0.0
Normal	50	0.2	0.2	0.1	0.1	0.1	0.1
	80	0.6	0.5	0.5	0.5	0.5	1.0
	20	0.0	0.0	-0.1	-0.1	-0.1	-0.1
Pos.	50	0.1	0.1	0.0	0.0	0.0	-0.1
skewed	80	0.6	0.5	0.4	0.4	0.4	0.7
	20	0.0	0.0	0.0	0.0	0.0	0.1
Neg.	50	0.1	0.1	0.1	0.1	0.0	0.2
skewed	80	0.6	0.6	0.7	0.7	0.8	1.9

The results show that censoring has almost no effect on the estimates. The percentage difference to the means of the measure in censored scenarios are less than 1 *%*, except in one case where the censoring proportion is more than 80 *%*. Even in this scenario, i.e. negatively skewed covariate, the means (standard deviation) of sampling distribution of *TG*_*STD*_(*T*_6_) for 0 *%* and 80 *%* censoring conditions are 0.280 (SD: 0.022) and 0.285 (SD: 0.062), respectively – a practically negligible difference in means.

**Fig. 2:**
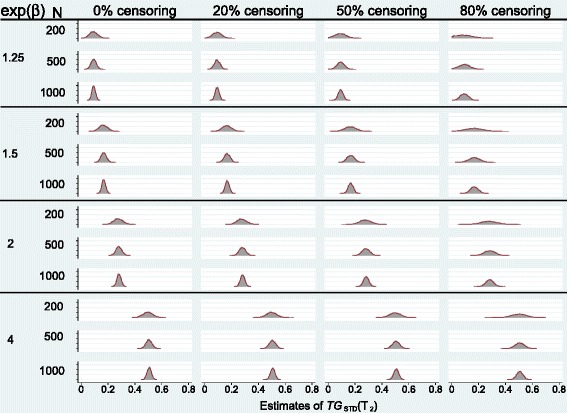
The sampling distribution of *T* *G*_*STD*_(*t*) by covariate effect *β*, sample size N, and censoring proportion. Number of replicates is 5,000 in all scenarios and the covariate, Z, is normally distributed

We conducted similar simulations with sample sizes of 200 and 1000 and different covariate effects which resulted in similar conclusions. However, the results showed that in general the percentage difference decreases as the sample size increases in all censoring proportions. As a measure of dispersion, we also calculated the standard deviation of sampling distribution of the measure (data not shown) in each experimental condition. The results showed that the dispersion of the measures decreases as sample size increases in all censoring conditions, as expected. To illustrate this, Fig. 2 presents the sampling distribution of *TG*_*STD*_(*T*_2_) for different covariate effects, sample sizes, and censoring proportions.

### Monotonicity and upper bound

The monotonicity property requires that *TG*_*STD*_(*t*) should increase with the size of covariate effect, i.e. |*β*|. In this section, we applied simulations to explore the means of both *TG* and *TG*_*STD*_ for a range of covariate effects where the distribution of survival time is exponential and the covariate is normally distributed.

**Fig. 3:**
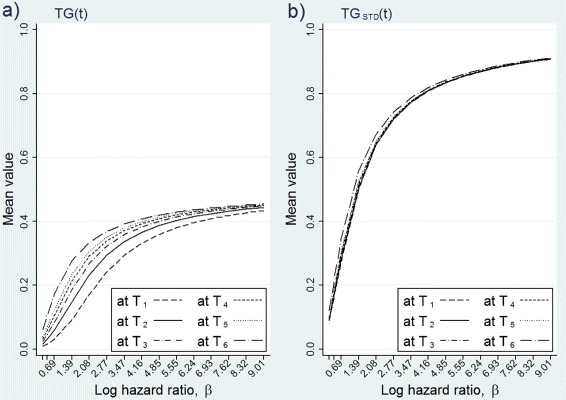
The means of *T* *G*(*t*) (**a**) and *T* *G*_*STD*_(*t*) (**b**) by the covariate effect in censored data. The covariate is normally distributed, random censoring (50 *%*) condition is used with a sample size of 500

The results are presented in graphs of Fig. 3. The figure shows the means of *TG*(*t*), left graph, and *TG*_*STD*_(*t*), right graph, at different time points by the covariate effect in the 50 *%* random censoring condition. As it is evident from the graphs, *TG*(*t*) increases with *β* and reaches a plateau of about 0.5; whereas *TG*_*STD*_(*t*) reaches values close to 1 for large *β*s. This accords with the finding of Bura and Gastwirth [9] for a logistic regression model where they showed that the upper bound of *TG* is less than or equal to 0.5. Finally, it is noticeable that the means of *TG*(*t*) at 6 time points differ for small values of *β*, but they converge as the covariate effect becomes stronger. The means of *TG*_*STD*_(*t*) are generally in agreement throughout the range of the covariate effect.

### Influential observations

In this section, we study the impact of extreme and outlier observations on *TG*_*STD*_(*t*) using simulations. We follow the definition of extreme and outlier observations as outlined in [1]: an extreme observation fits the underlying relationship between survival time and the covariate but it lies in the extremes of the (covariate) distribution, whereas the outlier observation does not fit the underlying relationship. In simulations, we generated survival times from an exponential distribution with one normally distributed covariate *N*(0,1) and covariate effect of *β*=0.69, with each replicated data set of size 200. Each data set was contaminated with a single extreme or outlier observation according to the procedure described in [1].

**Fig. 4:**
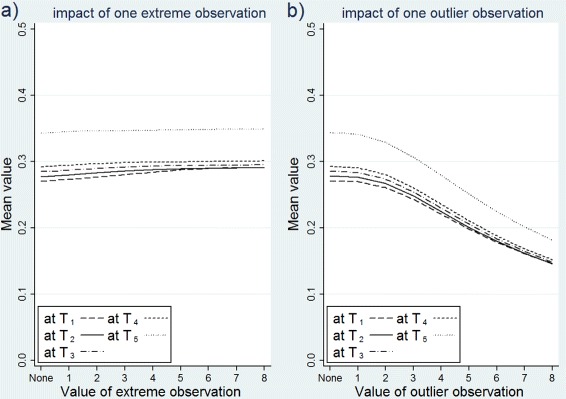
The impact of one extreme or outlier observation on the mean of *T* *G*_*STD*_(*t*). **a** one extreme observation (left); **b** one outlier observation (right). The covariate is normally distributed, random censoring condition, sample size = 200, and 50 *%*

The results are presented through graphs in Fig. 4. The graphs show the means of *TG*_*STD*_(*t*) by the value of the outlying covariate observation. For example, 4 in the *X* axis represents the condition where one observation’s covariate, *Z*∼*N*(0,1), is replaced with 4, and the corresponding value in the *Y* axis represents the mean of *TG*_*STD*_(*t*). The mean of *TG*_*STD*_(*t*) in the uncontaminated data is represented with ‘none’ on the *X* axis. The left graph in Fig. 4 shows the impact of one extreme covariate observation, and the right graph show the impact of one outlier covariate observation at 5 different time points. In the graphs, if a measure is resistant to extreme and outlier observations, its mean would not change in the presence of such observations. In other words, we expect a horizontal line across the *X* axis if the measure is resistant to such observations. The graphs indicate that the means of *TG*_*STD*_(*t*) are robust against the extreme observations, but they decrease rapidly as the outlier observation becomes more severe in all time points.

### Non-proportional hazard and time-dependent covariates

We carried out simulations to investigate the performance of *TG*_*STD*_(*t*) under non-proportional hazards (non-PH) and time-dependent covariate effect in a two arm trial setting. We used the Weibull distribution to generate time-to-event data and obtained the corresponding shape and scale parameters for the distribution of time-to-event data in each arm from IPASS (Iressa Pan-ASia Study) trial [24] - we used the same method as in [25] to estimate these parameters.

IPASS is a phase 3, two arm trial of previously untreated patients in East Asia who had advanced pulmonary adenocarcinoma (lung cancer) [24]. The main results from IPASS are summarized in Mok *et al.* [24]’s Figure two, which shows the distribution of time-to-event in each arm as Kaplan–Meier curves. Their Figure two(A) (i.e. Figure two, Panel A) shows that the progression-free survival curves cross at approximately 5.7 months, thus showing extreme non-PH. As it has been shown in [25], Weibull distributions with the following scale and shape parameters provide a good fit to the (censored) survival times in the two treatment arms: control arm parameters, scale = 0.35 and shape = 1.72; and experimental arm parameters, scale = 0.10 and shape = 1.08. We used these parameters to generate the survival times in the two groups. We truncated the time to event at 20 months to resemble the follow-up pattern in Mok *et al.* [24]’s Figure two(A).

**Fig. 5:**
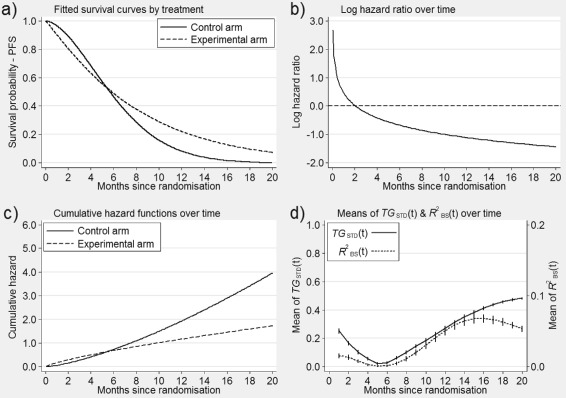
**a** Fitted survival curves by treatment group for the IPASS trial - simulated data. **b** Log hazard ratio over time. **c** Cumulative hazard functions by treatment. **d** The means of sampling distributions of *T* *G*_*STD*_(*t*) and $R_{\textit {BS}}^{2}(t)$ from 1 to 20 months - vertical lines represent the standard deviations in each scenario

The fitted survival curves by treatment arm are shown in Fig. 5(a). The curves cross at approximately the median survival time, which is about 5.7 months in each arm. We chose the same sample size as that of the IPASS trial, i.e. *n*=1127, with equal allocation ratio. We fitted a flexible parametric survival model [25] on the log cumulative hazard scale - see Additional file 2 for the details on the fitted model. Figures 5(b) and (c) show the fitted log hazard ratio and the cumulative hazard functions for the two arms. We then evaluated *TG*_*STD*_(*t*) from 1−20 months with 5,000 replications in each scenario. The results are summarised in Fig. 5(d). The mean (standard deviation) of the sampling distribution of *TG*_*STD*_(*t*) is 0.25(SD: 0.03) at 1 month, but it decreases towards 0 as the survival curves of the two arms converge. The mean reaches its minimal value of 0 where the survival curves cross at about 5.7 months. At this time point there is no separation/discrimination between the two groups and the mean value of *TG*_*STD*_(*t*) reflects this. It then increases as the survival curves diverge again until about 16 months, but it is starting to level off after this time point. Our results also showed a similar pattern for the mean of $ R_{\textit {BS}}^{2}(t)$ for the first 16 months; although the means of $ R_{\textit {BS}}^{2}(t)$ are much smaller throughout - see the right *y*-axis in Fig. 5(d). None of $R_{\textit {PM}}^{2}$, ${R_{D}^{2}}$, and ${\rho _{W}^{2}}$ can be used to meaningfully evaluate the performance of the model in this setting.

### Impact of categorisation of covariates

In this section, we study the impact of categorisation of covariates on *TG*_*STD*_(*t*). We carried out simulations to explore its performance when the continuous prognostic factors such as age and weight are categorised. Royston *et al.* [26] explained the dangers of dichotomisation of continuous covariates in the context of regression modelling, with the conclusion that it is an unnecessary practice for statistical analysis. They also showed that it will reduce both the amount of prognostic information and power, resulting in a reduction in the predictive ability of the fitted model.

In our simulations, the (conditional) distribution of survival times were exponential. The covariate was normally distributed as *N*(0,1) with an effect of exp(*β*)=4. We progressively categorised the covariate into *j*=2,...,20 categories by its quantiles. In each scenario, we computed percentiles corresponding to percentages 100∗*k*/*j* for *k*=1,2,...,*j*−1. For example, the categorisation of the covariate into 10 different groups requires that the 10th, 20th,..., 90th percentiles be computed.

To compare the performance of the measures with those of other proposed measures of predictive ability, we also carried out similar simulations for $ R_{\textit {PM}}^{2}$, ${R_{D}^{2}}$, ${\rho _{W}^{2}}$, and $R_{\textit {BS}}^{2}(t)$, which have been recommended by Choodari-Oskooei *et al.* [1, 3] for general use. $R_{\textit {PM}}^{2}$, ${R_{D}^{2}}$, and ${\rho _{W}^{2}}$ summarise the predictive ability for the entire follow-up period, whereas $ R_{\textit {BS}}^{2}(t)$ is time-dependent and changes over the follow-up period. $ R_{\textit {BS}}^{2}(t)$ is based on the (modified) Brier score [14]. Both $ R_{\textit {PM}}^{2}$ and ${R_{D}^{2}}$ are (monotonic) functions of the variance of the prognostic index of the model, whereas ${\rho _{W}^{2}}$ is based on the expected likelihood (entropy) under the full and null models - see [1, 3] for their formula and further details.

**Fig. 6:**
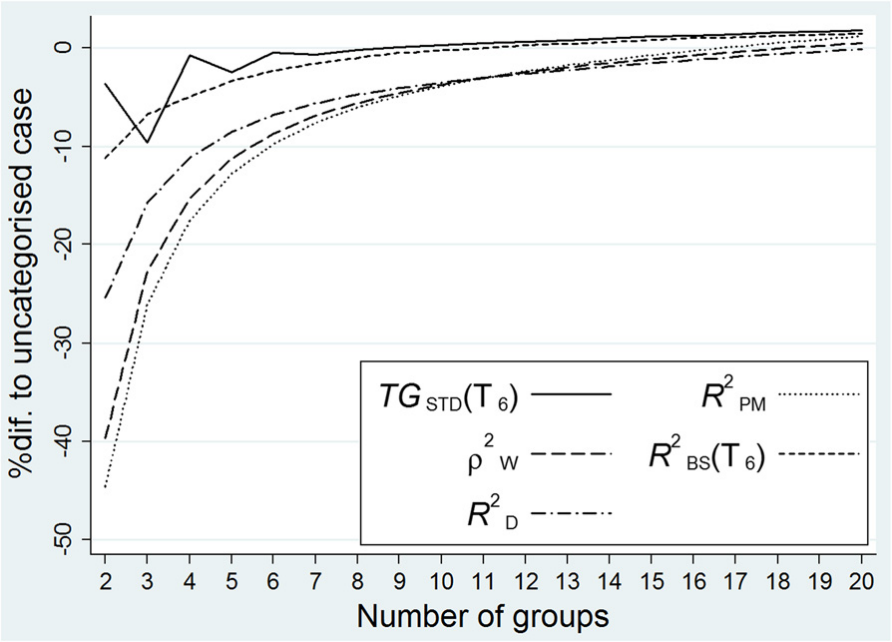
The impact of categorization on *T* *G*_*STD*_(*t*), $R_{\textit {PM}}^{2}$, $ {R_{D}^{2}}$, ${\rho _{W}^{2}}$, and $R_{\textit {BS}}^{2}(t)$ - *T* *G*_*STD*_(*t*) and $ R_{\textit {BS}}^{2}(t)$ were evaluated at *T*_6_. The percentage difference to the means of the measures in the uncategorised scenario plotted on the vertical axis against the number of groups (horizontal axis). In the uncategorised scenario, i.e. in the true model, the means of *T* *G*_*STD*_(*t*), $R_{\textit {PM}}^{2}$, $ {R_{D}^{2}}$, ${\rho _{W}^{2}}$, and $R_{\textit {BS}}^{2}(t)$ were 0.56, 0.54, 0.54, 0.64, and 0.33, respectively

The percentage difference between the means of the measures in the categorised scenario and the corresponding means of the measures in the uncategorised condition are displayed in Fig. 6. In general, a proportion of prognostic information is lost through grouping, which should be reflected in the estimates of the measures. As Fig. 6 demonstrates the loss of prognostic information is much more pronounced in the estimates of $ R_{\textit {PM}}^{2}$, ${R_{D}^{2}}$, and ${\rho _{W}^{2}}$. Except *TG*_*STD*_(*t*), all the other measures monotonically increase as the number of groups increases and reach values very close to the mean of the measures in the true model for more than 7 groups. The unexpected fluctuations in the means of *TG*_*STD*_(*t*) in less than 5 groups is due to the impact of integration under the predictiveness curve in these scenarios, which is a step function. In general, the loss of prognostic information is relatively small in both *TG*_*STD*_(*t*) and $R_{\textit {BS}}^{2}(t)$.

### Applications

In this section we present the results of investigation on the application of the measures to data sets from 5 disease areas. The estimates of the measures (with 95 *%* bootstrap confidence intervals) are displayed in Table 3. We also compare the estimates of *TG*_*STD*_(*t*) with those of $R_{\textit {PM}}^{2}$, ${R_{D}^{2}}$, $\rho _{W}^{2}$, and $R_{\textit {BS}}^{2}(t)$. The data sets are from: *i*) breast cancer (Schumacher *et al.*, [21]); *ii*) lymphoma (Rosenwald *et al.*, [27]); *iii*) primary biliary cirrhosis (PBC) (Fleming and Harrington, [28]); *iv*) renal cancer (Ritchie *et al.*, [29]); and *v*) prostate cancer (Byar and Green, [30]). All data sets are in the public domain - see Additional file 2.

**Table 3:** The estimates of *T* *G*_*STD*_(*t*), $R_{\textit {PM}}^{2}$, ${R_{D}^{2}}$, ${\rho _{W}^{2}}$, and $R_{\textit {BS}}^{2}(t)$, including 95 % bootstrap confidence intervals from 1000 replicates, in real data sets at 3 time points. The time points *T*_1_, *T*_2_, and *T*_3_ at which *T* *G*_*STD*_(*t*) and $R_{\textit {BS}}^{2}(t)$ are evaluated in all data sets are the 25th, 50th, and 75th quantile of the follow-up period, i.e. the time to the last event, in each study. Therefore, *T*_1_, *T*_2_, and *T*_3_ are different in each study

	Est. *T* *G*_*STD*_(*t*) at 3 time points						Est. $R_{\textit {BS}}^{2}(t)$ at 3 time points		
Study	$\widehat {{TG}}_{\textit {STD}}{(T}_{1}{)}$	$ \widehat {{TG}}_{\textit {STD}}{(T}_{2}{)}$	$\widehat {{TG} }_{\textit {STD}}{(T}_{3}{)}$	$\widehat {{R}}_{\textit {PM}}^{2}$	$ \widehat {{R}}_{D}^{2}$	$\widehat {{\rho }}_{W}^{2}$	$ \widehat {{R}}_{\textit {BS}}^{2}{(T}_{1}{)}$	$\widehat {{R }}_{\textit {BS}}^{2}{(T}_{2}{)}$	$\widehat {{R}}_{\textit {BS}}^{2} {(T}_{3}{)}$
Breast	0.32	0.33	0.35	0.27	0.28	0.36	0.12	0.16	0.20
cancer	(0.27-0.37)	(0.28-0.38)	(0.30-0.40)	(0.21-0.35)	(0.21-0.35)	(0.29-0.47)	(0.07-0.18)	(0.10-0.21)	(0.14-0.25)
Lymphoma	0.28	0.31	0.36	0.23	0.23	0.32	0.16	0.22	0.24
	(0.16-0.40)	(0.18-0.44)	(0.21-0.50)	(0.11-0.42)	(0.11-0.40)	(0.15-0.53)	(0.02-0.24)	(0.05-0.34)	(0.07-0.38)
PBC	0.58	0.62	0.56	0.56	0.65	0.60	0.38	0.47	0.47
	(0.52-0.65)	(0.54-0.70)	(0.50-0.62)	(0.48-0.65)	(0.55-0.74)	(0.53-0.68)	(0.19-0.52)	(0.38-0.58)	(0.34-0.57)
Renal	0.34	0.37	0.41	0.27	0.26	0.33	0.24	0.27	0.19
cancer	(0.28-0.40)	(0.31-0.42)	(0.36-0.46)	(0.21-0.36)	(0.20-0.33)	(0.27-0.42)	(0.16-0.31)	(0.21-0.34)	(0.11-0.26)
Prostate	0.22	0.24	0.26	0.13	0.13	0.18	0.06	0.11	0.10
cancer	(0.17-0.27)	(0.19-0.29)	(0.21-0.32)	(0.09-0.20)	(0.09-0.21)	(0.13-0.27)	(0.02-0.10)	(0.06-0.15)	(0.05-0.14)

Multivariable prognostic models based on the Cox PH model have already been developed for the above data sets. We applied the measures to these models to compare their performance. The first two data sets have been analysed extensively by Choodari-Oskooei *et al.* [1, 3]- see Additional file 2 for further details on the data sets, prognostic factors included in each study, and the summary of fitted models.

Table 3 shows the estimates of *TG*_*STD*_(*t*) and $R_{\textit {BS}}^{2}(t)$ at 3 time points, together with the estimates of $R_{\textit {PM}}^{2}$, ${R_{D}^{2}}$, and ${\rho _{W}^{2}}$. The 3 time points at which *TG*_*STD*_(*t*) and $ R_{\textit {BS}}^{2}(t)$ are evaluated in all data sets are the 25th, 50th, and 75th quantile of the follow-up period (the time to the last event) in each study. We emphasise that in practice a clinically motivated time point should be chosen. In all studies, the point estimates of *TG*_*STD*_(*t*) mildly increase with time, and are markedly higher than those for $ R_{\textit {BS}}^{2}(t)$. In some data sets, the estimates are within close range of those for $R_{\textit {PM}}^{2}$ and ${R_{D}^{2}}$.

**Fig. 7:**
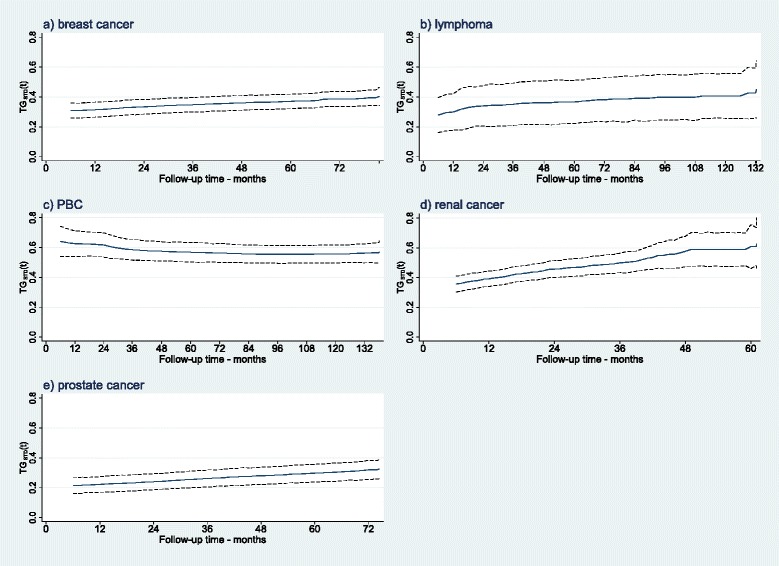
Estimates of TG_*STD*_(*t*) over the entire follow-up period in each study. **a** breast cancer study; **b** lymphoma data; **c** PBC study; **d** renal cancer data; and **e** prostate cancer. The solid lines represent the point estimates, and the dashed lines are the 95 % bootstrap confidence intervals with 1,000 replicates in each experimental condition

As stated before, the advantage of *TG*_*STD*_(*t*) over $R_{\textit {PM}}^{2}$, ${R_{D}^{2}}$, and ${\rho _{W}^{2}}$ is its ability to assess how the model’s predictive performance changes over time. Figure 7 shows the estimates of *TG*_*STD*_(*t*) over the entire follow-up in each study, together with the 95 *%* bootstrap confidence intervals. It can be concluded that the predictive ability of the models for breast cancer, PBC, and (to some extent) lymphoma studies relatively remain constant over the follow-up period.

## Discussion

In this paper, we described the predictiveness curve, and extended the (standardised) total gain statistic to a survival model. We carried out comprehensive simulation studies assessing its performance with respect to the criteria that a good measure of predictive ability should possess.

### Summary of our findings

Both *TG*(*t*) and *TG*_*STD*_(*t*) are based on the predictiveness curve. In simple terms, the predictiveness curve is a plot of the rank-ordered predicted survival probabilities versus the cumulative percentile for each predicted survival probability. The plot, therefore, illustrates the distribution of estimated risk (or survival probability predictions) in the population under study. The results of our empirical studies showed that both *TG*(*t*) and *TG*_*STD*_(*t*) are an increasing function of the covariate effect, and are independent of random censoring. Our results also showed that both measures are affected by the distribution of the prognostic factor in a survival model.

Our findings indicate that *TG*_*STD*_(*t*) is an increasing function of time in a multivariable regression model where the distribution of the PI is roughly Gaussian, whereas *TG*(*t*) increases with time until a certain time point and decreases afterwards towards zero in maximal survival times. This accords with the behaviour of the (modified) Brier score suggested by Graf *et al.* [14] for the survival models [3], and that of $R_{\textit {BS}}^{2}(t)$. The trend of *TG*(*t*) over time indicate that (for the models we studied) discrimination in survival probability predictions is minimal at the time origin as well as maximal time points, but it reaches its maximum around the median of the underlying distribution of survival time. We emphasise that the time points where both *TG*(*t*) and *TG*_*STD*_(*t*) are evaluated should be clinically relevant. We favour the use of *TG*_*STD*_(*t*) over *TG*(*t*) since it is in a similar scale to those of other *R*^2^-type statistics. *TG*(*t*) can easily be obtained from the estimate of *TG*_*STD*_(*t*) if the Kaplan-Meier estimate of survival probability at time *t*, $\widehat {\pi }_{0}(t)$, is also reported.

### Properties of *TG*_*STD*_(*t*)

An important property of *TG*_*STD*_(*t*) is that, unlike some of the proposed *R*^2^-type measures [1], it always lies between 0 and 1. Another advantage of *TG*_*STD*_(*t*) is its extendability to other types of survival model, including parametric survival models [25, 31]. The only underlying assumption in both measures is that the predictiveness curve *R*(*υ*;*t*) should be a monotonic function of the prognostic index in the model. Unlike $R_{\textit {BS}}^{2}(t)$ and other proposed predictive accuracy measures [3] (which assess the ability of the model to predict the outcome of interest at the individual level), *TG*_*STD*_(*t*) is a measure that quantifies the amount of prognostic information for a group of patients as it does not directly compare the individuals’ predicted risk probabilities with their actual outcomes. For a survival model, $R_{\textit {PM}}^{2}$, ${R_{D}^{2}}$, and ${\rho _{W}^{2}}$ also quantify the amount of prognostic information for a group of patients. They, however, provide an overall measure of predictive ability for the entire follow-up period. In this respect, *TG*_*STD*_(*t*) has an advantage over these measures because, inherently, it is a function of time and can be used to compare studies with different follow-up periods. But, its (perceived) downside is that it does not provide a unique value for a given model. One possible solution is to define an integrated version of *TG*_*STD*_(*t*) over the entire follow-up period - similar to the integrated $R_{\textit {BS}}^{2}(t)$ proposed by Graf *et al.* [14].

Bura and Gastwirth [9] showed that *TG* is normally distributed for a logistic regression model and developed a formula for its variance. The results of our simulations showed that the sampling distribution of *TG*(*t*) is also (asymptotically) normal (e.g. see Fig. 2). Based on the (large sample) asymptotic distribution of *TG*, Bura and Gastwirth [9] developed an asymptotic formula for its variance - see Additional file 1. In principle, the formula can be adopted (with some amendments) for use in a survival model. However, since it is based on the normal approximation to the probability of having an event by time *t*, i.e. *π*_0_(*t*), it might not provide a good approximation if the (effective) sample size is small and *π*_0_(*t*) is near 0 or 1. We, therefore, propose bootstrap resampling to construct confidence intervals.

Finally, most *R*^2^-type measures proposed for survival models lack the intuitive interpretation of *R*^2^ in linear regression as explained variation. *TG*_*STD*_(*t*) is not an exception in this regard. Therefore, further research is needed to explain these measures (and their properties) in a way that is easily accessible to practical researchers.

### Relationship to other measures

We have shown that $R_{\textit {Pepe}}^{2}(t)$ (i.e. *TG*_*STD*_(*t*) with squared error loss) is the model-based, i.e. parametric, version of $R_{\textit {BS}}^{2}(t)$. Therefore, they are asymptotically equivalent if the model is correctly specified. It can be argued that the assumption of correctly specified model may not be entirely feasible in practice. Nonetheless, the smaller variance in (the estimates of) $R_{\textit {Pepe}}^{2}(t)$ makes it an appealing choice - i.e. the classic bias versus variance trade-off. Further research is required to study the trade-off between the bias and variance of these estimators in a series of simulations based on real datasets. For a logistic regression model, the relationships between the predictiveness curve *R*(*υ*;*t*), the *c*-statistic, and reclassification measures have been established [10]. For a survival model, however, this is a topic for further research.

## Conclusions

Our studies showed that the total gain measure performed well with respect to our criteria. It can also be applied to a broad class of survival models. Overall, we believe that it can be recommended as a measure to quantify the predictive ability in survival models.

## Additional files

Additional file 1
**In this document** ${\mathbf {R_{\textit {BS}}^{2}(T^{\ast })}}$ **,** ${\mathbf {R_{\textit {PM}}^{2}}}$ **, and** ${\mathbf {{R_{D}^{2}}}}$ **are formally defined and the relationship between** ${\mathbf {R_{\textit {Pepe}}^{2}}}$ **and** ***V***_***B***_ **is explored.** The asymptotic formula for the variance of *TG* is also presented.

Additional file 2
**The multivariable prognostic models applied to the data sets in** Applications **, as well as the flexible parametric model applied in** Non-proportional hazard and time-dependent covariates **, are presented.**
