# Supplementary material for: The extension of total gain (TG) statistic in survival models: properties and applications
Source: BMC Med Res Methodol. 2015 Jul 1;15:50. doi: 10.1186/s12874-015-0042-x (PMC4486698; doi:10.1186/s12874-015-0042-x)
Supplement: Additional file 1 — In this document ${\mathbf {R_{\textit {BS}}^{2}(T^{\ast })}}$RBS2(T∗) , ${\mathbf {R_{\textit {PM}}^{2}}}$RPM2 , and ${\mathbf {{R_{D}^{2}}}}$RD2 are formally defined and the relationship between ${\mathbf {R_{\textit {Pepe}}^{2}}}$RPepe2 and V B is explored. The asymptotic formula for the variance of TG is also presented. [file 12874_2015_42_MOESM1_ESM.pdf]

Additional file 1

In this document first $R^2_{BS}(T^*)$, R^2_{PM}, and R^2_D are formally defined. Next, the relationship between R^2_{Pepe} and V_B is explored. Finally, the asymptotic formula for the variance of TG as developed by Bura and Gastwirth (2001) [1] is presented.

Graf *et al.* $R^2_{BS}(T^*)$

$R^2_{BS}(T^*)$ is based on quantifying the average prediction error (over patients) at each time point using a quadratic loss function. This error is known as a Brier score and weights are used to compensate for the loss of information due to censoring. The conditional prediction error at time T^*, $PE_Z\left(T^*\right)$, is defined as

$$BS\left(T^*|Z\right) = \int_Z E\left[\left(I\left(T > T^*\right) - S(T^*|Z)\right)^2\right] dF_Z(Z)$$

where $I\left(T > T^*\right) \in \{0, 1\}$ is the individual survival status at time T^* and $S(T^*|Z)$ is the predicted survival probabilities from the model with covariate vector Z. The marginal prediction errors at time T^*, $BS\left(T^*\right)$, is defined similarly, but $S(T^*|Z)$ is replaced with $S(T^*)$, i.e. the predicted survival probability under the null model. Graf *et al.* [2] defines

$$R^2_{BS}(T^*) = \frac{BS\left(T^*\right) - BS\left(T^*|Z\right)}{BS(T^*)}$$

Kent and O'Quigley R^2_{PM}

This measure is based on the definition of R^2 for linear regression and seeks to quantify the proportion of variation in the outcome explained by the predictors in the model

$$R^2_{PM} = \frac{Var(\beta' Z)}{Var(\beta' Z) + 1.645}$$

where 1.645 is the variance of the error term in an equivalent Weibull model.

Royston and Sauerbrei R^2_D

This measure is similar to R^2_{PM} but is based on the authors' own D statistic, a measure of prognostic separation described in [3]. That is,

$$R^2_D = \frac{D^2/k^2}{D^2/k^2 + 1.645}$$

where $k = \sqrt{(8/\pi)}$. The ratio D^2/k^2 is an estimator of the variance of the PI, provided that the PI is Normally distributed.

Relationship between R^2_{Pepe} **and Schemper's** V_B

Using the notations of Section 2.3 in Bura and Gastwirth [1], $\int_0^1 (R(v) - \pi_0)^2 dv$ in Equation 4 (of the main manuscript) can be written as $n^{-1} \sum_i (\pi_i|Z - \pi_0)^2$ where $\pi_i|Z$ is the

model-based risk prediction for individual i and π_0 is the average risk probability. For a binary outcome Y with mean π_0 the variance is $n^{-1}\sum_i (Y_i - \pi_0)^2 = \pi_0(1-\pi_0)$ which is the denominator of Equation 4. Furthermore, the numerator in V_B can be written as $n^{-1}\sum_i (Y_i - \pi_0)^2 - n^{-1}\sum_i (Y_i - \pi_i|Z)^2 = n^{-1}\sum_i (\pi_i|Z - \pi_0)^2$ which is the numerator in Equation 4.

Asymptotic formula for the variance of TG

For a logistic regression model:

$$E(Y|Z=z) = \pi|Z = \frac{\exp(\alpha + \beta z)}{1 + \exp(\alpha + \beta z)}$$

Bura and Gastwirth [1] developed the following asymptotic formula for the variance of TG:

$$\begin{aligned} \sigma^2_{TG} &= 4\{F_*^2 \pi_0(1-\pi_0) + \pi_0 F_*(1-F_*) + F_*^2 \pi_0(\pi_c - \pi_0) \\ &+ \frac{\int_{-\infty}^{z^*} \pi^2(z)dF(z)}{F_*} - \frac{\left(\int_{-\infty}^{z^*} \pi(z)dF(z)\right)^2}{F_*^2} + 2\pi_0(1-F_*)\int_{-\infty}^{z^*} \pi(z)dF(z)\} \end{aligned}$$

where $z^* = (\log \frac{\pi_0}{1-\pi_0} - \alpha)/\beta,\ F_* = \int_{-\infty}^{z^*} dF(z)$, and $\pi_c = E(Y|Z \leq z^*) = \int_{-\infty}^{z^*} \pi(z)dF(z)/F_*$.

References

[1] E. Bura and J. L. Gastwirth. The binary regression quantile plot: Assessing the importance of predictors in binary regression visually. *Biometrical Journal*, 43(1):5–21, 2001.

[2] E. Graf, C. Schmoor, W. Sauerbrei, and M. Schumacher. Assessment and comparison of prognostic classification schemes for survival data. *Statistics in Medicine*, 18:2529–2545, 1999.

[3] B. Choodari-Oskooei, P. Royston, and M. K. B. Parmar. A simulation study of predictive ability measures in a survival model I: Explained variation measures. *Statistics in Medicine*, 31(23):2627–2643, 2012.
