# Supplementary material for: The extension of total gain (TG) statistic in survival models: properties and applications
Source: BMC Med Res Methodol. 2015 Jul 1;15:50. doi: 10.1186/s12874-015-0042-x (PMC4486698; doi:10.1186/s12874-015-0042-x)
Supplement: Additional file 2 — The multivariable prognostic models applied to the data sets in Applications , as well as the flexible parametric model applied in Non-proportional hazard and time-dependent covariates , are presented. [file 12874_2015_42_MOESM2_ESM.pdf]

Additional file 2

In this document the multivariable prognostic models applied to the data sets in Section 3.8, as well as the flexible parametric model applied in Section 3.6, are presented. Table 1, below, shows model χ^2 and degrees of freedom, skewness and kurtosis of the prognostic indices resulting from the fitted regression models in Section 3.8. The outcome variables and prognostic factors are outlined below.

Breast cancer study

The data set is available from:

http://portal.uni-freiburg.de/imbi/Royston-Sauerbrei-book/index.html#datasets

The outcome variable is progression-free survival. The prognostic factors in the multivariable model are: age, tumour grade (`tg`), number of positive lymph nodes (`pln`), progesterone receptor (`pr`), and hormonal therapy (`ht`). The PI of this model is [1]

$$1.74\,\texttt{age}^{-2} - 7.82\,\texttt{age}^{-0.5} + 0.52\,(\texttt{tg} \geqslant 2) - 1.98\,\exp\,(-0.12\,\texttt{pln}\,) - 0.058\,(\texttt{pr}+1)^{0.5} - 0.394\,\texttt{ht}.$$

Lymphoma study

Data can be obtained from:

http://llmpp.nih.gov/DLBCL/

The outcome is overall survival. The prognostic factors included in the model are: A three-level "International Prognostic Index" (`IPI`) based on both clinical and pathological factors is currently used for risk stratification of patients with aggressive lymphoma (low risk: IPI 0-1 ($\texttt{ipi}_1$), intermediate: IPI 2-3 ($\texttt{ipi}_2$) and high: IPI 4-5 ($\texttt{ipi}_3$)) and a genetic factor (`gene17`)[2]. The PI of the model is

$$0.718\,\texttt{ipi}_2 + 1.042\,\texttt{ipi}_3 + 0.719\,\texttt{gene17}$$

with the reference group being the patients with the lowest risk, i.e. $\texttt{ipi}_1$.

PBC study

Data can be obtained from:

http://lib.stat.cmu.edu/datasets/pbc

We used 312 individuals that participated in the clinical trial part of the study. The outcome is overall survival. The prognostic factors in the model are: age, oedema (`oedema`), log albumin (`albumin`), log bilirubin (`bilir`), and log prothrobin time (`ptime`) - see [3]. The PI of the model is

$$0.03\,\texttt{age} + 0.78\texttt{oedema} - 3.05\ln(\texttt{albumin}) + .88\ln(\texttt{bilir}) + 3.02\ln(\texttt{ptime})\ .$$

Renal cancer study

Data can be obtained from:

http://portal.uni-freiburg.de/imbi/Royston-Sauerbrei-book/index.html#datasets

The outcome is overall survival. The prognostic factors in the model are: A three-level WHO performance status, haemoglobin (`haem`), white cell count (`wcc`), interferon-α treatment (`treat`), and time from metastasis to randomisation (`tmt`). The PI of the model is

$$0.29\texttt{WHO}_2 + 0.89\texttt{WHO}_3 - 0.21\texttt{haem} + 0.07\ln(\texttt{wcc}) - 0.33\texttt{treat} + 0.04\texttt{tmt}$$

with the reference group being the patients with the lowest WHO performance status, i.e. $\texttt{WHO}_1$.

Prostate cancer study

The data is available here:

http://biostat.mc.vanderbilt.edu/wiki/Main/DataSets?CGISESSID=dd39d78b86e36698ce66ccf4ea20d125

The outcome is overall survival. The prognostic factors in the model are: age; standardised weight (`wt`); acid phosphates (`ap`); haemoglobin (`hg`); and size of primary tumour (`sz`); performance status (`pf`); and history of cardiovascular disease (`hx`). The PI of the model is

$$0.02\texttt{age} - 0.01\texttt{wt} + 0.06\ln(\texttt{ap}) - 0.01\texttt{hg} + 0.02\texttt{sz} + 0.38\texttt{pf} + 0.47\texttt{hx}.$$

Flexible parametric model applied in Section 3.6

In Section 3.6, the following (flexible) parametric survival model [4] on the log cumulative hazard scale was fitted:

$$\ln H(t|z) = \gamma_0 + \gamma_1 \ln(t) + \delta_1 \ln(t) z + \beta z$$

where $H(t|z)$ is the cumulative hazard function, t is the (censored) survival time, z is the binary covariate, and γ_0, γ_1, $\delta_1$and β are the model parameters. In this model, the corresponding time-dependent hazard ratio, $HR(t)$, is

$$\ln HR(t) = \ln(\frac{\gamma_1 + \delta_1}{\gamma_1}) + \delta_1 \ln(t) + \beta.$$

Table 1: Summary of the models applied to the data sets in Section 3.8, model χ^2 and degrees of freedom, skewness and kurtosis of the prognostic indices resulting from the fitted regression models.

Study	Model χ^2	*d.f.*	Sample Size	% Censored	PI Skewness	PI Kurtosis
Breast cancer	153.11	6	686	56	0.21	3.88
Lymphoma	17.64	3	73	34	-0.19	2.11
PBC	199.13	5	312	40	0.98	3.60
Renal cancer	132.69	6	347	7	0.81	4.96
Prostate cancer	77.41	7	506	30	0.40	3.05

References

[1] W. Sauerbrei and P. Royston. Building multivariable prognostic and diagnostic models: transformation of the predictors by using fractional polynomials. *Journal of the Royal Statistical Society (Series A)*, 162:71–94, 1999. Corrigendum: *Journal of the Royal Statistical Society (Series A)*, 165:399–400, 2002.

[2] D. Dunkler, S. Michiels, and M. Schemper. Gene expression profiling: Does it add predictive accuracy to clinical characteristics in cancer prognosis? *European Journal of Cancer*, 43:745–751, 2007.

[3] J. F. Lawless. *Statistical Models and Methods for lifetime Data.* Wiley and Sons, New York, 2 edition, 2002.

[4] P. Royston and P. C. Lambert. *Flexible Parametric Survival Analysis Using Stata: beyond the Cox model.* Stata Press, 2011.
